# Immunogenicity in African Green Monkeys of M Protein Mutant Vesicular Stomatitis Virus Vectors and Contribution of Vector-Encoded Flagellin

**DOI:** 10.3390/vaccines6010016

**Published:** 2018-03-19

**Authors:** Marlena M. Westcott, Jason Smedberg, Matthew J. Jorgensen, Shelby Puckett, Douglas S. Lyles

**Affiliations:** 1 Department of Microbiology and Immunology, Wake Forest School of Medicine, Winston Salem, NC 27101, USA; 2 Department of Biochemistry, Wake Forest School of Medicine, Winston Salem, NC 27101, USA; jasonsmedberg@gmail.com (J.S.); spuckett@wakehealth.edu (S.P.); dlyles@wakehealth.edu (D.S.L.); 3 Department of Pathology, Section of Comparative Medicine, Wake Forest School of Medicine, Winston Salem, NC 27101, USA; mjorgens@wakehealth.edu

**Keywords:** vesicular stomatitis virus, vaccine, vector, flagellin, nonhuman primate, African green monkey

## Abstract

Recombinant vesicular stomatitis virus (VSV) is a promising platform for vaccine development. M51R VSV, an attenuated, M protein mutant strain, is an effective inducer of Type I interferon and dendritic cell (DC) maturation, which are desirable properties to exploit for vaccine design. We have previously evaluated M51R VSV (M51R) and M51R VSV that produces flagellin (M51R-F) as vaccine vectors using murine models, and found that flagellin enhanced DC activation and VSV-specific antibody production after low-dose vaccination. In this report, the immunogenicity of M51R vectors and the adjuvant effect of virus-produced flagellin were evaluated in nonhuman primates following high-dose (10^8^ pfu) and low-dose (10^5^ pfu) vaccination. A single intramuscular vaccination of African green monkeys with M51R or M51R-F induced VSV-specific, dose-dependent humoral immune responses. Flagellin induced a significant increase in antibody production (IgM, IgG and neutralizing antibody) at the low vaccination dose. A VSV-specific cellular response was detected at 6 weeks post-vaccination, but was neither dose-dependent nor enhanced by flagellin; similar numbers of VSV-specific, IFNγ-producing cells were detected in lymph node and spleen of all animals. These results indicate that virus-directed, intracellular flagellin production may improve VSV-based vaccines encoding heterologous antigens by lowering the dose required to achieve humoral immunity.

## 1. Introduction

Vaccines based on a live, attenuated vesicular stomatitis virus (VSV) platform are being developed for a wide range of infectious diseases and cancer [1,2]. For example, recombinant VSV-based vaccines against Ebola virus and HIV, which express Ebola glycoprotein or HIV gag, respectively, have demonstrated safety and efficacy in clinical trials [3,4]. Attenuation of VSV for the purpose of engineering safe vaccine vectors has been achieved by substituting or mutating the VSV glycoprotein gene (G) which is essential for viral infectivity [5,6], by changing the order of the 5 genes that comprise the VSV genome leading to insufficient production of viral proteins at key steps in the life cycle [7,8], or by a combination of the two approaches [9,10,11]. An alternative attenuation strategy is to genetically inactivate the ability of VSV to suppress host antiviral responses. Mutations at position 51 of the viral matrix (M) protein render the virus unable to suppress host antiviral responses but do not compromise its ability to express viral gene products [12]. VSV with M51R and ΔM51 mutations in M protein are robust inducers of Type I interferon (IFN) and IFN-induced genes and activate multiple subtypes of murine and human dendritic cells to produce T cell-activating costimulatory molecules and pro-inflammatory cytokines [13,14,15,16,17]. Further, M51R VSV induces robust immune responses in mice [18,19]. These properties could be exploited and refined to develop the M51R VSV strain as a live vaccine vector for delivery of heterologous antigens.

Bacterial flagellin has been widely investigated as an adjuvant for non-living vaccines, including killed and protein nanoparticle influenza virus vaccines [20,21], and the fusion protein vaccine for pneumonic plague, Flagellin/F1/V, which has completed preclinical evaluation in nonhuman primates and a Phase I clinical trial [20,22,23]. The adjuvant activity of flagellin as a component of killed, subunit and particle-based vaccines is mediated largely through its interaction with TLR5 [24], an extracellular TLR expressed by hematopoietic and epithelial cells [25,26]. In addition to signaling via TLR5, bacterial flagellin also has the capacity to stimulate cells through cytosolic pattern recognition receptors [26], an activity for which less information is available in the context of vaccines. We have reported that activation of murine and human dendritic cells by M51R VSV can be further enhanced by engineering the virus to express the *Salmonella enterica fliC* gene (M51R-F) [19,27]. In this context, VSV-directed flagellin production enhances dendritic cell activation through interaction with the cytosolic NOD-like receptor C4 (NLRC4) inflammasome complex [26,27,28]. In addition, we demonstrated that flagellin produced by M51R VSV improved VSV-specific IgG production in response to low-dose intranasal vaccination of mice [19]. Based on these results, and to inform further development of M51R VSV as a vaccine vector for delivery of heterologous antigens, we sought to evaluate the adjuvant potential of cytosolic flagellin using a nonhuman primate vaccination model. The goal of the studies in the current report was to evaluate the ability of the two vectors to induce adaptive immune responses in adult African green monkeys. Specifically, we measured VSV (vector)-specific immune responses after one intramuscular injection with M51R or M51R-F at low or high dose. The results demonstrate an effective dose range for the induction of primary antibody responses, which were enhanced by the expression of flagellin at the low vaccination dose. In contrast, the generation of VSV-specific interferon γ-secreting cells in spleen and lymph nodes was neither dose-dependent nor enhanced by the presence of flagellin.

## 2. Materials and Methods

### 2.1. Virus

Recombinant M protein-mutant VSV (M51R VSV) and M51R VSV that constitutively produces flagellin (M51R-F VSV) as a result of introduction of the *fliC* gene encoding flagellin from *Salmonella enterica,* serovar Enteritidis, between the viral M and G genes, were described previously [19,27]. Briefly, the *fliC* gene was inserted as an independent transcription unit between the M and G genes of the VSV genome. The production of flagellin in the intracellular compartment following infection of permissive cells with M51R-F VSV was confirmed by western blot [27]. For the flow cytometry-based neutralizing antibody assay, an M51R VSV strain that constitutively produces enhanced green fluorescent protein (M51R-eGFP) was used [29]. All viruses, including wild-type VSV (Indiana serotype, Orsay strain, used for the VSV-specific antibody ELISA) were propagated in BHK cells, and titers were determined using a BHK cell plaque assay.

### 2.2. Animals, Vaccination and Tissue Collection

Sixteen female, adult African green monkeys (AGM, vervet subspecies, *Chlorocebus aethiops sabaeus*) were used for the study. AGM were bred and housed at the Wake Forest School of Medicine Primate Center. Animals ranged in age from 8.5 to 15.5 years, and in weight from 4.2 to 7.89 kg, and were cared for and handled according to guidelines of the National Institutes of Health Guide for the Care and Use of Laboratory Animals and the Wake Forest Institutional Animal Care and Use Committee (protocol A14-184). Animals were chosen for the study based on low pre-existing immunity to VSV or flagellin as measured by the presence of antibodies in pre-immune serum. All procedures were performed on animals that were anesthetized using ketamine (10–15 mg/kg, i.m.). After collecting pre-immune blood samples by femoral venipuncture using standard heparin vacutainers, animals were split into 4 cohorts (consisting of 4 animals/cohort) and vaccinated in the right bicep muscle with 0.5 mL of purified live, recombinant M51R VSV (M51R) or M51R VSV expressing the flagellin gene (M51R-F) at 2 different doses (1 × 10^5^ PFU, low dose and 1 × 10^8^ PFU, high dose). At weeks 1, 2 and 4 post-vaccination, blood was collected, processed to obtain plasma, and frozen at −80 °C. At 6 weeks post-vaccination animals were euthanized (15–20 mg/kg ketamine, i.m. followed by 60–100 mg/kg pentobarbital, i.v.), and blood and tissue samples were harvested. For the current study, spleens and axillary lymph nodes (right draining and left contralateral) were processed to obtain single cell suspensions devoid of red blood cells, frozen and stored in liquid N_2_.

### 2.3. Cells

VSV-permissive mouse EL4 cells [30] were cultured in DMEM supplemented with 10% fetal calf serum (FCS) and 2mM glutamine. For experiments with frozen AGM cells (spleen, lymph node), the cells were thawed and subject to a recovery period at 37 °C, 5% CO_2_ for 2 h in RPMI supplemented with 10% FCS, 2 mM glutamine, 100 Units/mL penicillin, 100 ug/mL streptomycin, and 2-mercaptoethanol (2ME, (5 × 10^−5^ M)). The cells were then used as antigen presenting cells or as responder cells in IFNγ Elispot assays, as specified below.

### 2.4. ELISA for Detection of VSV-Specific Antibody

Nunc MaxiSorp ELISA plates were coated with 0.2 µg/well purified VSV (Orsay strain) in sodium carbonate coating buffer (pH 9.6). Wells without virus served as a negative control. Plates were blocked with phosphate buffered saline containing 0.05% Tween 20 (PBST) + 5% skim milk. Plasma samples were serially diluted in PBST (wash buffer). Antibodies specific for monkey IgG (Fitzgerald, 43R-1G020HRP, Acton, MA, USA) and IgM (LifeSpan Bioscience, LS-C61207, Seattle, WA, USA) directly conjugated to horseradish peroxidase (HRP) were used for detection. Plates were developed with 1-step Turbo TMB-ELISA substrate (Thermo Scientific #34022, Waltham, MA, USA), and the absorbance at 450 nm was measured on a BMG Labtech POLARstar Omega microplate reader (Cary, NC, USA). The absorbance for each sample was calculated by subtracting the absorbance obtained from corresponding wells that were not coated with virus. The concentration of IgG or IgM was determined from standard curves prepared with monkey IgG (#017-0102-0001) and IgM (#017-0107) Rockland Immunochemicals (Pottstown, PA, USA).

### 2.5. Affinity ELISA

IgG affinity was measured by thiocyanate elution according to a published protocol [31] by modifying the VSV-specific antibody ELISA as follows. Six-week plasma samples were diluted to achieve a mid-curve OD_450_ value relative to the monkey IgG standard curve. After a 2 h incubation of duplicate plasma samples on VSV-coated and blocked wells, the wells were washed 5 times with PBST and 100 uL of buffer containing sodium thiocyanate (NaSCN, starting at 2 M and diluted 2-fold serially to 0.06 M) was added for 15 min. The wells were again washed 5 times with PBST before proceeding with the secondary antibody incubation (30 min) and substrate steps (15 min). The % IgG remaining bound to the wells after NaSCN treatment relative to controls not treated with NaSCN, was calculated as follows: (OD_450_ + NaSCN)/(OD_450_ − NaSCN) × 100. The data were expressed as the concentration of NaSCN that removed 50% of bound IgG (EC_50_), and reported as the mean EC_50_ ± SD for each cohort.

### 2.6. Neutralizing Antibody Assay

A flow cytometry-based virus neutralization assay was developed according to a published protocol [32], with modifications. Heat-inactivated (56 °C for 1 h) plasma samples from vaccinated monkeys were serially diluted in DMEM supplemented with 2% heat-inactivated FCS in a 96 well round-bottom plate. An equal volume of M51R-eGFP (1.5 × 10^5^ pfu/well) was added to the wells, and samples were incubated for 1 h at 37 °C, 5% CO_2_ to allow for antibody binding to the virus. In the next step, 100 uL of pre-incubated sample was added to an equal volume of EL4 cells (3 × 10^6^/mL in DMEM supplemented with 7% heat-inactivated FCS and 2 mM glutamine) in a 96-well round bottom dish. After a 5 h, 37 °C, 5% CO_2_ incubation to allow for virus infection, EL4 cells were harvested from the wells, washed and fixed with 4% paraformaldehyde. Samples were acquired on a BD FACSCalibur Flow Cytometer and analyzed with FlowJo software. The neutralizing antibody titer was defined as the dilution of plasma that inhibited the infection of EL4 cells by 50%, which was determined using GraphPad Prism software. Controls consisted of EL4 cells with no virus added, and EL4 cells infected with M51R-eGFP in the absence of plasma (for maximum infection level). The infection protocol was calibrated such that approximately 30% of the cells were infected with M51R-eGFP in the absence of neutralizing antibodies.

### 2.7. IFNγ ELISPOT Assay

To measure the cellular immune response against VSV vectors, an IFNγ ELISPOT assay kit was used according to manufacturer’s instructions (Mabtech, Inc. #3420M-2H, Cincinnati, OH, USA). Cells from the left axillary lymph node (LLN) were used as antigen presenting cells (APC), and autologous cells from the right (draining) axillary lymph node (RLN) or spleen were used as responding cells. LLN cells were infected at a concentration of 1.0 × 10^6^ cells/mL with M51R VSV at MOI = 50, or were mock-treated, for 18 h at 37 °C, 5% CO_2_. Mock- or virus-infected APC were irradiated using an MDS Nordion Gammacell 1000 Elite (Ottawa, ON, Canada) irradiation source (2000R). APC (2.5 × 10^5^/well) were mixed with an equal volume (100 μL) of autologous responder cells (2 × 10^5^/well). Cells were cultured for 48 h in ELISPOT plates (Millipore, #MAIPSWU10, Darmstadt, Germany) coated with anti-IFN-γ capture antibody. Spot-forming cells were detected using biotinylated secondary antibody and True Blue peroxidase substrate (KPL #50-78-02, Milford, MA, USA) according to manufacturer instructions. Spots were photographed and quantitated using an ImmunoSpot system (Cellular Technology, Ltd., Shaker Heights, OH, USA).

### 2.8. Murine Vaccination Study

Female 8 weeks old C57BL/6 mice (Charles River, Wilmington, MA, USA) were immunized intranasally with 5 × 10^5^ PFU of M51R or M51R-F. VSV-specific T cell responses were measured in spleen on days 6, 8, 10 and 12. Splenocytes were re-stimulated in vitro with peptides corresponding to an immunodominant N protein epitope for CD8^+^ T cells [33] or G protein epitope for CD4^+^ T cells [34]. Intracellular cytokine staining was performed using fluorescently-tagged antibodies against murine CD4, CD8, CD44, IFNγ, TNFα, IL-4, IL-17A, with appropriate isotype controls (BD Biosciences, San Jose, CA, USA). Fluorescence data were acquired using a BD Canto instrument, and data were analyzed using FlowJo software. Mouse experiments were performed according to a protocol approved by the Wake Forest School of Medicine Animal Care and Use Committee (#A13082).

### 2.9. Statistical Analysis

Antibody data were analyzed as either the antibody titer (neutralizing antibody) or plasma antibody concentration (virus-specific IgM and IgG). Data were analyzed by two-factor analysis of variance (ANOVA), with time and cohort as the two factors, using SigmaStat for Windows version 3.5 software (Systat Software, Inc., San Jose, CA, USA). Post-hoc multiple comparisons were made by the Holm-Sidak and Bonferroni methods with an alpha level of *p* < 0.05 after correction for multiple comparisons. Cellular immune response data were analyzed by one-way ANOVA for comparison of multiple cohorts.

## 3. Results

### 3.1. Anti-VSV Antibody Response

**Figure 1:**
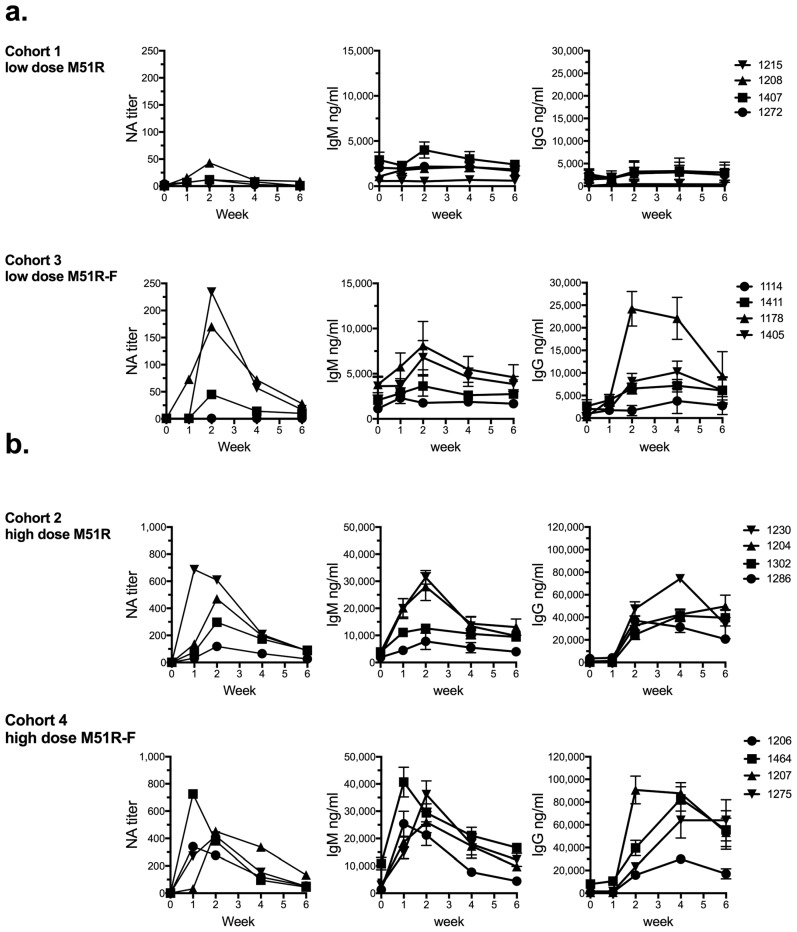
Humoral immune response. Plasmas were analyzed before vaccination (week 0, pre-immune) and at weeks 1, 2, 4 and 6 post-vaccination for anti-VSV antibodies. Neutralizing antibody (NA) titer (left panel) was defined as the dilution of serum that inhibited infection of mouse EL4 cells with M51R-eGFP by 50%. IgM (middle panel) and IgG (right panel) levels are expressed in ng/mL (mean ± SD, triplicate values), measured by ELISA with extrapolation from standard curves. Data for low dose (**a**) and high dose (**b**) vaccinations with M51R and M51R-F are shown. Note scale differences for low and high dose data. Statistical significance was determined by two-factor analysis of variance with cohort and time as the two factors. For all three antibody types, statistical significance of *p* < 0.05 (after correction for multiple comparisons) was obtained for comparisons of cohort 1 versus cohort 3 (low dose M51R vs. M51R-F), cohort 1 versus cohort 2 (low dose M51R vs. high dose M51R), and cohort 3 versus cohort 4 (low dose M51R-F vs. high dose M51R-F), but not for cohort 2 versus cohort 4 (high dose M51R vs. high dose M51R-F). The data shown represent 1 of 2 analyses with similar results performed on plasma from each animal.

The cohorts for the study were as follows: C1: low dose M51R (1 × 10^5^ PFU); C2: high dose M51R (1 × 10^8^ PFU; C3: low dose M51R-F (1 × 10^5^ PFU); C4: high dose M51R-F (1 × 10^8^ PFU). For each animal and time point, plasma samples were tested to quantify VSV-specific neutralizing antibodies (NA), IgM and IgG from low-dose- (Figure 1a) and high dose- (Figure 1b) vaccinated animals. The NA titer was defined as the dilution of plasma that inhibited infection of a permissive cell line with VSV-eGFP by 50%, as determined by flow cytometry analysis (Figure 1, left panels). Two of 4 animals in the low-dose M51R cohort 1 (1407 and 1208) produced NA that was ≥4-fold above pre-immune levels, with titers of 12 and 43, respectively. In comparison, low dose vaccination with M51R-F (cohort 3) induced NA in 3 of 4 animals (1411, 1178 and 1405). The NA titers for those animals were 45, 170 and 234, respectively. The increase in NA titer in the low dose M51R-F relative to M51R-vaccination group was significant as determined by two-factor ANOVA with time and cohort as the two factors. In contrast to low dose vaccination, animals that received high dose M51R (cohort 2) or M51R-F (cohort 4) produced NA well above pre-immune levels, with the exception of one animal (1286) in cohort 2 that was a low responder relative to the others. In the high dose groups, there was no significant difference in the NA response after vaccination with M51R-F relative to M51R. NA levels peaked between 1 and 2 weeks, and by 6 weeks had declined for all animals in all cohorts.

Anti-VSV IgM production (Figure 1, middle panels) was low (1407 and 1208) or undetectable (1272 and 1215) relative to pre-immune levels in the low dose M51R group (cohort 1), consistent with NA results. In the low dose M51R-F group (cohort 3), IgM responses were greatest in AGM 1178 and 1405, similar to NA results (Figure 1a). Again, the difference in IgM responses in the low dose M51R-F as compared to the low dose M51R group was statistically significant. Animals vaccinated with high dose M51R (cohort 2) produced IgM at levels 3-13-fold above pre-immune at 2 weeks (Figure 1b). Animal 1286 was again the low responder in cohort 2. The presence of flagellin (cohort 4) did not improve IgM responses at the high dose (4-19-fold increase above pre-immune).

Low dose M51R vaccination (cohort 1) induced minimal or undetectable IgG responses (<2-fold above pre-immune) (Figure 1a, right panels) similar to results for IgM and NA. In the low dose M51R-F-vaccinated group (cohort 3) however, 3 of 4 animals produced IgG that was ≥4-fold above pre-immune levels at peak (1114, 4-fold; 1405, 5-fold; 1178, 74-fold). IgG responses in the low dose M51R versus M51R-F-vaccinated groups were significantly different.

In contrast to low dose IgG results, high dose vaccination with M51R-F did not enhance IgG responses relative to the high dose M51R group (Figure 1b, right panels). This finding is consistent with NA and IgM responses showing no effect of flagellin at the high vaccination dose. For the high dose groups, IgG peaked at 2 or 4 weeks, and was still elevated in some cases at 6 weeks post-vaccination. In addition, the data indicate that the humoral response to both VSV vectors was dose-dependent. There was a significant difference in low dose as compared to high dose antibody responses for each vector (compare cohort 1 to cohort 2 (M51R) and cohort 3 to cohort 4 (M51R-F), noting scale differences). The response to vaccination by criteria of all 3 parameters measured (NA, IgM and IgG) was consistent for individual animals. However magnitude of response did not necessarily correlate, in part due to the VSV-specific NA assay reflecting the humoral response to G protein, while the ELISAs reflect the response to all viral proteins.

As an additional measure of flagellin adjuvant potential, IgG affinity was measured. The rationale for this experiment was based on the report of a vaccine comprised of *Pseudomonas aeruginosa* outer membrane proteins fused to flagellin that induced antigen-specific IgG with high affinity and protective function in mice and AGM [35,36]. We therefore subjected anti-VSV IgG in 6 week post-vaccination plasma samples to analysis using a modified VSV ELISA protocol as described in Materials and Methods. Using this assay, relative IgG affinities can be compared by measuring resistance to elution from antigen-coated wells by sodium thiocyanate (NaSCN) treatment [31]. The data are expressed as NaSCN concentration necessary to remove 50% of bound IgG from the wells after 15 min of treatment [EC_50_]. This analysis performed on 6 week plasma from each of the 16 monkeys yielded no significant differences among cohorts, (mean EC_50_ ± SD): Low dose M51R: 1.00 ± 0.70 M; Low dose M51R-F: 0.84 ± 0.33 M; High dose M51R: 1.20 ± 0.05 M; High dose M51R-F: 1.17 ± 0.14 M (*p* > 0.05, one way analysis of variance). Therefore by this criteria, expression of flagelin by the M51R vector did not modulate affinity maturation of the anti-VSV IgG response.

### 3.2. Anti-VSV Cellular Response

**Figure 2:**
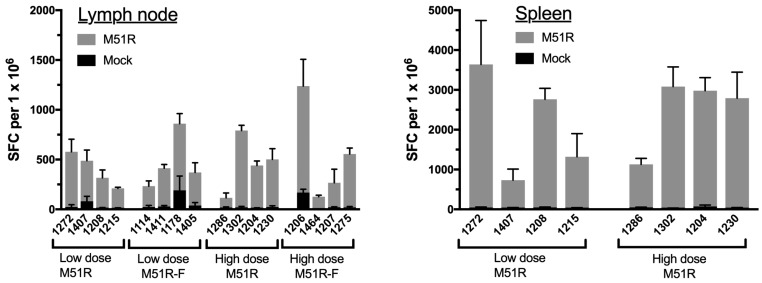
Cellular immune response in lymph node and spleen. Cells from the 6 week right axillary lymph nodes were re-stimulated with autologous APC infected with M51R VSV, and IFNγ production was measured by ELISPOT (gray bars). The number of spot-forming cells (SFC) per 10^6^ lymph node responder cells is shown for each animal (mean ± SD, triplicate values). Data from responder cells restimulated with mock-infected APC are shown by the black bars. The same experiment was done with spleen cells (right panel) from low and high dose M51R-vaccinated animals (cohorts 1 and 2, respectively). For both lymph node and spleen experiments, no significant differences between the 4 cohorts were found by ordinary one-way ANOVA (*p* > 0.05).

IFNγ ELISPOT assay was used to measure the cellular response to in vitro restimulation with live VSV at 6 weeks post-vaccination. Antigen-presenting cells (APC) were prepared by infection of left axillary lymph node cells with M51R VSV for 24 h followed by X-irradiation prior to addition of responder cells from the draining (right) lymph node. Results are expressed as the number of spot forming cells (SFC) per 10^6^ responder cells (Figure 2). Lymph node cells from all animals produced IFNγ in response to re-stimulation with M51R VSV-infected APC (gray bars), while IFNγ production was minimal when APC were mock-infected (black bars) The magnitude of the IFNγ response was similar among the 4 cohorts; all animals produced a response that ranged from 100–900 SFC/10^6^ cells regardless of vaccine dose or the presence of flagellin. Monkey 1286 from the M51R high-dose group (cohort 2), was a low-responder as measured by IFNγ-producing cells, consistent with its low responder status with regard to antibody production (Figure 1). Using spleen rather than lymph node as responder cells, a dose-independent production of IFNγ was again observed (Figure 2). Therefore, by the criteria of IFNγ production after VSV-restimulation, cellular responses of similar magnitude were generated after vaccination with live M51R at 10^5^ and 10^8^ PFU. Further, flagellin encoded by the M51R-F vector did not enhance the response relative to M51R vector without adjuvant.

## 4. Discussion

This study presents data using nonhuman primates to evaluate dosage and adjuvant function of flagellin when produced by M51R VSV, which has been previously evaluated in mice as a live virus vaccine vector [19]. The results contribute new information with regard to this vaccine platform. First, a single intramuscular vaccination of adult AGM with 10^8^ PFU of M51R VSV induced substantial VSV-specific humoral responses as measured by VSV-specific IgM, IgG and neutralizing antibodies. Second, a VSV-specific cellular response was detected in lymph node and spleen at 6 weeks post-vaccination, as measured by IFNγ production after in vitro restimulation with VSV. Third, the VSV-specific humoral but not cellular immune response was dose-dependent. Fourth, M51R-directed intracellular flagellin production enhanced anti-VSV antibody production in the context of low dose vaccination only (10^5^ PFU), and did not affect the generation of a cellular response at either vaccination dose.

**Figure A1:**
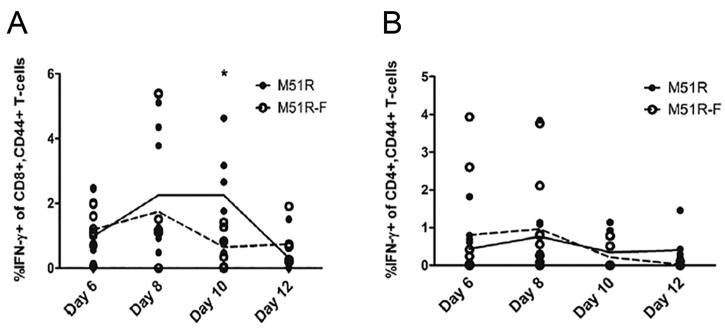
Murine CD8^+^ T cell activation after vaccination with M51R and M51R-F vaccine vectors. Eight week-old mice were immunized intranasally with 5 × 10^5^ PFU of M51R or M51R-F, and VSV-specific CD8^+^ T cell responses in the spleen were measured at days 6, 8, 10 and 12 post-vaccination. Splenocytes were re-stimulated in vitro with peptides corresponding to an immunodominant N protein epitope for CD8^+^ T cells or G protein epitope for CD4^+^ T cells. Intracellular cytokine staining and flow cytometry were used to measure T cell activation. Points shown are the percentage of cytokine-producing T cells within the CD8^+^ CD44 high splenic population for individual mice. The lines (solid, M51R; dotted, M51R-F) indicate the mean of 9 mice (6 days and 8 days) or 6 mice (10 days and 12 days) per vaccination group. (**A**) IFNγ-producing cells (**B**) TNFα-producing cells. No significant differences were found between the 2 groups at any time point except for day 10 (indicated by a *), in which vaccination with M51R induced a greater IFNγ response than did M51R-F (*p* < 0.05, Student’s *t*-test).

**Figure A2:**
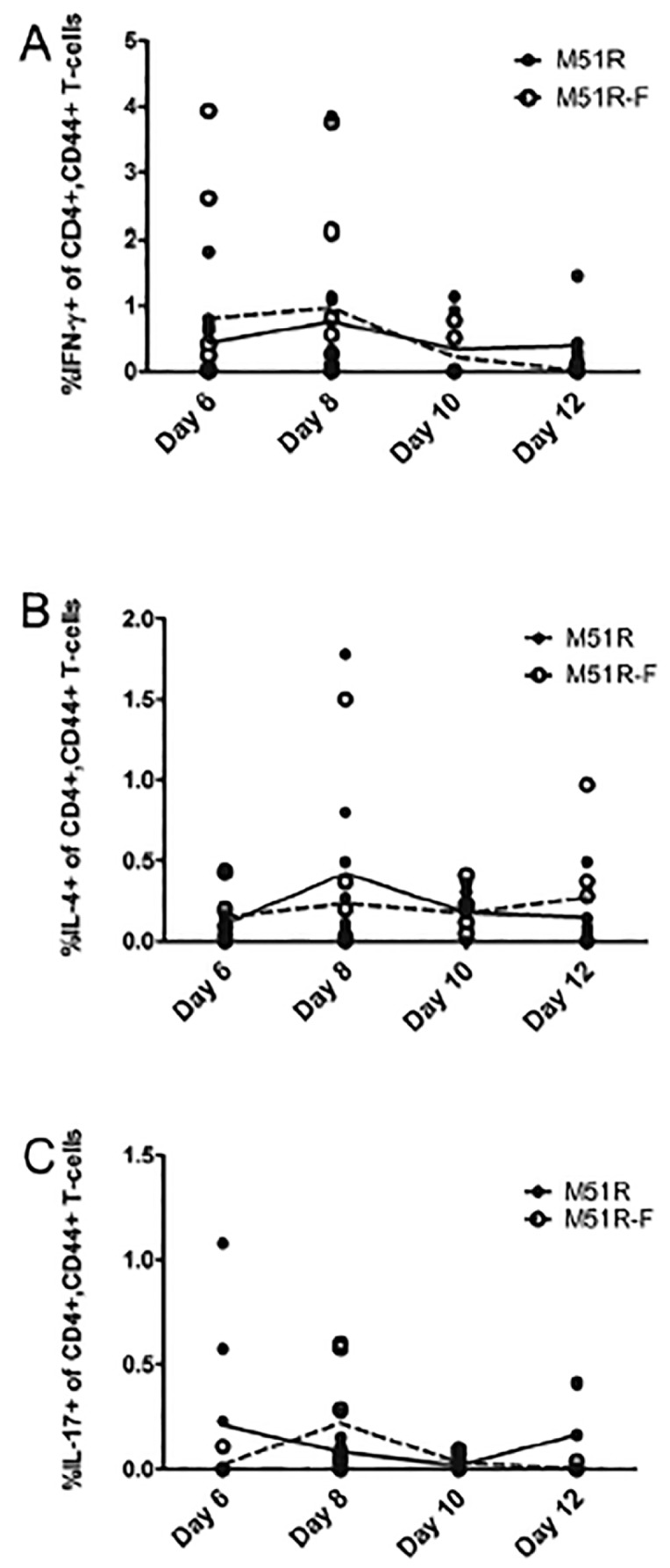
Murine CD4^+^ T cell activation after vaccination with rM51R and rM51R-F vaccine vectors. CD4^+^ T cell responses were measured as per CD8 T cell responses. Points shown are the percentage of cytokine-producing T cells within the CD4^+^ CD44 high splenic population for individual mice. The lines (solid, M51R; dotted, M51R-F) indicate the mean of 9 mice (6 days and 8 days) or 6 mice (10 days and 12 days) per vaccination group. (**A**) IFNγ-producing cells (**B**) IL-4-producing cells (**C**) IL-17A-producing cells. No significant differences were found between the M51R and M51R-F-vaccinated groups at any time point (*p* > 0.05, Student’s *t*-test).

Many studies to date have tested flagellin mixed with antigen, flagellin-antigen fusion proteins, or flagellin associated with noninfectious particles, all of which stimulate cells through TLR5 [20,21,22,25,37]. In contrast, flagellin produced by replicating VSV, as evaluated in this study, activates the intracellular NLRC4 inflammasome complex, which among other effects activates caspase-1 and IL-1β production by innate immune cells [27,28]. We observed a significant effect of M51R-driven flagellin production in augmenting antibody production at the low vaccination dose (10^5^ PFU), which is consistent with our previously published data using the same vectors to vaccinate mice via an intranasal route [19]. This finding is consistent with previous reports that when administered along with weak vaccines, IL-1β mediates enhanced immunogenicity and protection in several models of bacterial and viral infection, possibly through its capacity to augment T cell expansion and effector function [38,39]. With regard to our data, flagellin-driven IL-1β production may have augmented antibody production via the promotion of T cell help, or indirectly via the production of pro-inflammatory cytokines by infected innate immune cells. The current study was not designed to test the utility of M51R-encoded flagellin for inducing protective immunity in a pathogenic context. It remains possible that the inflammasome-stimulating activity of flagellin could augment protection against viral or bacterial infections for which IL1-β is a key component, such as influenza [40,41] and *Pseudomonas aeruginosa* [42]. With regard to the potential for a T cell-mediated mechanism driving enhanced humoral immunity at low vaccination dose, we used the same vectors in the murine intranasal vaccination model and did not find that flagellin enhanced primary VSV-specific T cell responses, as measured by cytokine production following in vitro re-stimulation of splenocytes with immunodominant G protein [34] and N protein [33] peptides, respectively (Appendix Figure A1 and Figure A2). The mechanism by which M51R-encoded flagellin improves humoral immunity in mice and AGM in the context of low dose vaccination, and the potential for M51R vectors to confer protective immunity will be the subject of future studies.

A VSV-specific cellular response, as measured by the number of IFNγ-producing cells in lymph node and spleen after in vitro re-stimulation, was detected in all animals at 6 weeks post-vaccination. Interestingly, the generation or maintenance of these cells did not depend on vaccine dose; similar numbers of IFNγ producing cells were detected in all animals in both spleen and lymph node, regardless of vaccine dose. Further, the presence of flagellin in the system (M51R-F-vaccinated animals) had no effect at either vaccination dose. The cells produced IFNγ in response to VSV-infected antigen presenting cells, with minimal cytokine produced in cultures that were stimulated with mock-infected APC (Figure 2), indicating that IFNγ production was indeed VSV antigen-driven. Dose independence of the cellular response could reflect a low viral antigen requirement for T cell activation. For example, replication defective (single cycle) viruses, including VSV, have been shown to induce CD8+ T cell responses of similar magnitude as their replication-competent counterparts [43,44]. Although we did not identify the cell type producing IFNγ in the ELISPOT assay, preliminary fractionation experiments indicated that IFNγ was produced by both CD3^+^ and CD3^−^ cells, suggesting that in addition to T cells, IFNγ-producing long-lived NK cells [45] or B cells [46] could contribute to the host response to M51R vectors in nonhuman primates. Given the importance of IFNγ in immune responses against intracellular pathogens and tumors, this possibility warrants future studies with the AGM model to phenotype the cells that produce IFNγ in response to M51R vectors. 

M protein mutant VSV is one of a number of attenuated VSV strains that have potential as vaccine vectors [9,47]. An advantage of the M protein-mutant is its capacity to undergo multiple cycles of replication in several subtypes of DC while preserving cell integrity and inducing maturation [13,14,15,17]. These properties would be expected to increase the availability of vector-encoded antigens for presentation to T cells and to activate other innate immune cells, as demonstrated for example in a DC-based cancer vaccine model [17]. Another advantage is the high capacity of M protein mutant VSV to induce production of Type I IFN, which supports the activation of functional T and B cell responses [48]. The significantly greater humoral immune response that we observed after high dose relative to low dose administration of M51R vectors could reflect a greater viral load and higher Type I IFN levels early after vaccination, which could in turn enhance VSV-specific B cell functionality as reported previously [49].

Further development of M51R and M51R-F as vaccine platforms raises several considerations. First, an assessment of efficacy in inducing an immune response against a cloned heterologous antigen (from an emerging virus or tumor, for example) is needed. Second, to more rigorously evaluate vector-directed flagellin production will require engineering the virus to efficiently produce both antigen and adjuvant. Third, a safety evaluation will be necessary. There is precedent for addressing these issues successfully [1]. The 11 kb VSV genome can tolerate up to 6 kb of additional sequence with modest effects on viral titer [50,51]. We’ve reported that expression of the 1.6 kb *fliC* gene as a separate transcription unit between the M and G genes did not affect viral titers [19]. One possible strategy going forward would be to engineer the antigen of choice as an independent transcription unit into the first position of the M51R and M51R-F genomes. This positional strategy has yielded high level heterologous gene expression in conjunction with virus attenuation [1,2]. The availability of a sensitive NHP model for safety testing [52] could be leveraged to assess the combined M protein and positional antigen placement strategies for attenuation.

## 5. Conclusions

This study demonstrates efficacy of 10^8^ PFU of M51R VSV for inducing substantial adaptive immune responses in the absence of a boost. This may be attributed in part to the robust response of DC to infection with M protein mutant VSV strains [13,14,19,27,53], the proinflammatory nature of which is key to the development of effective adaptive immune responses against viruses and cancer. Further, M51R-directed flagellin production enhances antibody responses in AGM vaccinated with as little as 10^5^ PFU of vector, raising the possibility that virus-directed intracellular flagellin production could improve the performance of M51R VSV as a vaccine vector by lowering the dose necessary to achieve humoral immunity against target antigens. These results form the basis for further development of M51R VSV as an alternative vaccine platform.
